# Experimental transmission of Stony Coral Tissue Loss Disease results in differential microbial responses within coral mucus and tissue

**DOI:** 10.1038/s43705-022-00126-3

**Published:** 2022-05-30

**Authors:** Naomi Huntley, Marilyn E. Brandt, Cynthia C. Becker, Carolyn A. Miller, Sonora S. Meiling, Adrienne M. S. Correa, Daniel M. Holstein, Erinn M. Muller, Laura D. Mydlarz, Tyler B. Smith, Amy Apprill

**Affiliations:** 1 Center for Marine and Environmental Studies, University of the Virgin Islands, St. Thomas, USVI USA; 2 Marine Chemistry and Geochemistry Department, Woods Hole Oceanographic Institution, Woods Hole, MA USA; 3 MIT-WHOI Joint Program in Oceanography/Applied Ocean Science and Engineering, Cambridge and Woods Hole, MA USA; 4 Department of Biosciences, Rice University, Houston, TX USA; 5 Department of Oceanography and Coastal Science, Louisiana State University, Baton Rouge, LA USA; 6 Mote Marine Laboratory, Sarasota, FL USA; 7 Department of Biology, University of Texas at Austin, Austin, TX USA

**Keywords:** Microbial ecology, Microbiology

## Abstract

Stony coral tissue loss disease (SCTLD) is a widespread and deadly disease that affects nearly half of Caribbean coral species. To understand the microbial community response to this disease, we performed a disease transmission experiment on US Virgin Island (USVI) corals, exposing six species of coral with varying susceptibility to SCTLD. The microbial community of the surface mucus and tissue layers were examined separately using a small subunit ribosomal RNA gene-based sequencing approach, and data were analyzed to identify microbial community shifts following disease acquisition, potential causative pathogens, as well as compare microbiota composition to field-based corals from the USVI and Florida outbreaks. While all species displayed similar microbiome composition with disease acquisition, microbiome similarity patterns differed by both species and mucus or tissue microhabitat. Further, disease exposed but not lesioned corals harbored a mucus microbial community similar to those showing disease signs, suggesting that mucus may serve as an early warning detection for the onset of SCTLD. Like other SCTLD studies in Florida, Rhodobacteraceae, Arcobacteraceae, Desulfovibrionaceae, Peptostreptococcaceae, Fusibacter, Marinifilaceae, and Vibrionaceae dominated diseased corals. This study demonstrates the differential response of the mucus and tissue microorganisms to SCTLD and suggests that mucus microorganisms may be diagnostic for early disease exposure.

## Introduction

Marine diseases are growing in magnitude and severity causing economic and biodiversity impacts on marine ecosystems [1–5]. Historically the role of individual microorganisms as causative agents has been the focus of marine disease studies, but it is increasingly recognized that microbiomes, assemblages of associated microorganisms, play a critical role in organismal health and immune function [6]. The tendency for microbial communities to remain stable when exposed to stressors, or undergo dysbiosis, the breakdown of microstructure and diversity, are emerging as important elements of stress and disease responses.

In the marine environment, specific diseases generally affect one species, and it is rare that similar disease signs are exhibited across diverse species [7, 8]. However, host fidelity appears less restrictive within diseases of scleractinian corals. Stony coral tissue loss disease (SCTLD) affects phylogenetically diverse Caribbean corals (>22 species) with distinct morphologies and growth rates [9]. Examining microbiome stability and dysbiosis associated with the onset of disease signs within a multi-species experimental framework provides an opportunity to uncover the characteristics of microorganisms or communities that may be involved in maintaining disease resilience.

Despite the toll SCTLD is having on Caribbean coral reefs [10–14], little is known about the microbial contributions to this disease. The diversity, species specificity, and variability of coral-microbial associations all contribute to the challenges of identifying a disease pathogen. Antibiotic treatment has been shown to halt progression of SCTLD, suggesting a bacterial origin [15, 16]. Spatial epidemiological models of disease spread have suggested that the pathogen(s) may be waterborne [17, 18]. Recently, evidence suggests that a virus may be associated with the disease [19, 20]. While there have been some bacterial taxa correlated with the disease in field-based surveys [21–23], no etiological agent(s) have been identified and no transmission studies have specifically examined coral microbiomes during SCTLD onset. Further, why some coral species are susceptible to SCTLD while others are not remains unclear but may be due to species-specific traits such as differential host gene expression, or symbiont (e.g., dinoflagellate endosymbionts of corals in the family Symbiodiniaceae) and microbiome characteristics, which ultimately affect the innate host immune function. Understanding SCTLD-associated microbiome dynamics in a species-specific, as well as coral microhabitat (mucus or tissue) framework is necessary to address this question.

Previous studies have found that coral “microhabitats” (e.g., mucus, tissue, skeleton) have distinct microbial communities [24, 25]. Further, differentiation between these microhabitats allows for a more targeted understanding of microbial dynamics upon disease acquisition. Mucus layers are found in a vast variety of organisms, from humans to cnidarians, yet the function of this layer is similar among species, in providing a protective barrier between the organism and the external environment. Mucus microbial communities tend to be influenced by both environmental and host factors [25], playing an important role in coral immunity as a physical barrier that traps and immobilizes pathogens [26]. Furthermore, this layer is inhabited by beneficial microbes that exclude pathogens from penetrating the mucus layer and thus infecting the coral tissue through both competition and the secretion of antibiotic substances [27, 28].

The microbes intimately associated with coral tissue (exclusive of mucus or skeleton) are less studied. Coral tissue is composed of three layers: epidermis, gastrodermis, and mesoglea, with Symbiodiniaceae symbionts located in the gastrodermis and microbial aggregates identified within epidermal and gastrodermal tissues of healthy corals [29, 30]. Histological evidence from Landsberg et al. [31] suggests that SCTLD first affects the Symbiodiniaceae, with lesions originating in the gastrodermis. Thus, focusing on identifying tissue-associated microbes may provide more specific information about potential causative agents or perhaps secondary or opportunistic pathogens responding to tissue sloughing.

Advances in human microbiome studies have shown that many stressors and disease result in microbial dysbiosis -- a shift to a microbial community that is detrimental to the organism’s health [32, 33]. This shift in the microbiome from mutualistic to dysbiotic can be seen as a reduction in beneficial microbes, an increase in pathogenic microbes, and both reductions and increases in microbial diversity [34–37]. For coral diseases, few have an identified etiological agent, and it is unclear if disease is caused by a single pathogen or rather a shift that occurs in the microbial community. This shift to a dysbiotic community may reduce the efficacy of the microbes in the mucus layer to protect the host and also may reduce the host’s ability to fight infection [33, 36] and may be seen as an increase in pathogens present in both the mucus and tissue. In a disease transmission study by Macknight et al. [33] the microbial community of white plague disease infected coral holobiont samples converged to a community with reduced microbial diversity that was dominated by pathogens. Further, the changes to the microbiome followed a species susceptibility pattern.

To understand coral mucus and tissue microbiome dysbiosis upon exposure to SCTLD, we conducted a laboratory-based transmission study with six coral species that vary in terms of their disease susceptibility, reported lesion progression rates in the field [38], and their representation of phylogenetic and ecological diversity. We hypothesized that both the mucus and tissue microbiomes of diseased corals would significantly differ from that of healthy colonies, and that diseased mucus and tissue would show different microbiome disease signatures. Further, we hypothesized that, upon disease acquisition, the most susceptible species would show greater microbial community similarities among affected colonies compared to the least susceptible coral species.

## Materials and methods

### Transmission experiment

**Fig. 1:**
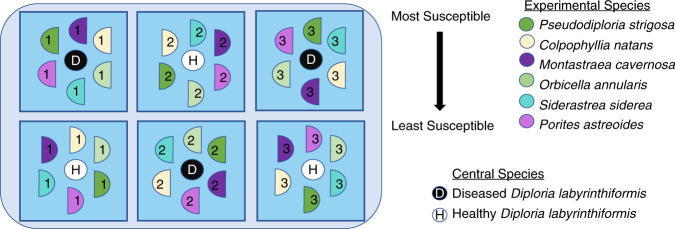
Schematic of the disease transmission experimental setup. Six coral species (listed from most to least susceptible and represented by different colors) were cut in half, allowed to acclimate, and then placed in either a control or treatment tank. Half circles with matching color and number indicate being matching halves from a colony. Squares represent the aquaria in which corals were kept within the larger flow through seawater table. Black and white round circles represent central colonies which were either collected from a disease-free site for the control group or a Stony Coral Tissue Loss Disease infected *D. labyrinthiformis* in the treatment group. Three healthy and three treatment aquaria are shown here, but there were eight total.

Colonies from six species of coral were exposed to either diseased or apparently healthy *Diploria labyrinthiformis* in a randomized mesocosm array (Fig. 1). Portions of this study, including disease prevalence and incidence, relative risk of lesion development, and lesion progression rates were previously reported in Meiling et al. [39]. Apparently healthy coral colonies were collected from Ruperts Rock, St Thomas, USVI (18° 19′ 39.6″ N 64° 55′ 33.5″ W) on 03/22 and 03/26/2019 following a roving survey of the site, which was conducted immediately prior to the collection to ensure that the site was free of SCTLD. Apparently healthy colonies that were approximately 25 cm × 25 cm (*n* = 8) of each of the six species (Fig. 1) were collected and transported in individual ziplock bags placed in a cooler to the University of the Virgin Islands Center for Marine and Environmental Studies to be used as ‘treatment’ colonies.

Diseased and healthy *D. labyrinthiformis* colonies (*n* = 8 of each) were collected for use as the central ‘donor’ colony in the diseased or control experiment treatments, respectively, on 04/03/2019 (one day prior to the experiment). The disease-free apparently healthy central colonies were collected from the same site as the experimental treatment colonies (Ruperts Rock) by one team of divers and were used for the ‘control’ treatments. To avoid contamination, a separate boat and dive team collected colonies of *D. labyrinthiformis* with active disease from the purported initial disease outbreak location, Flat Cay, St Thomas (18° 19′ 02.9″ N 64° 59′ 27.0″ W). Central diseased colonies were observed for 24 h before being used in experiments to confirm that lesions were expanding –indicating active disease-- prior to use as the experimental disease treatment. Throughout the experiment, all central diseased colonies showed active lesion expansion and no central apparently healthy colonies exhibited lesion development.

Each apparently healthy colony was separated into two fragments prior to one-week acclimation to allow for a fragment of each genet to be exposed to a coral colony showing signs of SCTLD and an apparently healthy control colony. Upon commencement of the transmission experiment on 04/04/2019, one half of the treatment fragment belonging to each species was arranged at equal distances around an actively diseased *D. labyrinthiformis* (treatment *n* = 8), while the other half of the treatment fragment was placed in a separate 26 l tank arranged in a similar manner except the central *D. labyrinthiformis* was an apparently healthy coral (control *n* = 8) (Fig. 1, modeled after Williams et al. [40]). Tanks were filled with seawater pumped from Brewer’s Bay that went through two different sediment settling tanks, pumped through a 20 µm pleated sediment filter that includes two rounds of UV sterilization (80 W and then 40 W). Tanks were placed among three outdoor, shaded seawater tables with chilled running seawater to maintain a constant temperature. Each tank was equipped with an individual air stone, 100% of water was changed daily, and tanks were randomly redistributed across the seawater tables daily.

Disease treatment fragments that developed lesions were monitored for at least 12 h to confirm that had an actively expanding lesion indicative of SCTLD. Corals with active lesions were removed from the experimental tank along with the corresponding genet fragment from the paired control tank. Corals were briefly removed from the water long enough to collect mucus using sterile cotton flocked swabs (Hydraflock, Puritan, Guilford ME, USA) and an approximately 8 cm^2^ colony fragment was collected at the lesion using a hammer and chisel for tissue microbiome analysis. The experiment was ended after 10 days and treatment corals that did not develop disease were removed from the experiment and sampled along with their corresponding control fragment. The swabs were placed in a cryovial and coral fragments (tissue + skeleton) were stored at −80 °C for later molecular analysis.

### Comparative field samples

To allow for comparison to tank samples, diseased coral fragments of *Orbicella annularis* and *Pseudodiploria strigosa* (n = 4) were collected in the field on 04/07/2019 from the same site as diseased experimental corals (Flat Cay). Approximately 2 cm^2^ coral fragments were collected by SCUBA using a hammer and chisel and brought to the surface in a whirl-pak bag filled with seawater and swabbed along the lesion for mucus (field mucus). The fragments (field tissue) were then transported in a cooler back to UVI and both field mucus and field tissue were stored at −80 °C. Coral samples were also taken on 02/11 and 02/13/2020 of *Montastraea cavernosa* and *Colpophyllia natans*, from Buck Island (18° 27′ 88.3″ N, −64° 89′ 83.3″ W) and Black Point (18° 34′ 45″ N, −64° 98′ 59.5″ W) reefs surrounding St. Thomas, as described in Becker et al. [22]. The outbreak of SCTLD at Buck Island was reported in January 2020, one year after the disease was first reported in the USVI at Flat Cay and Black Point. A 10 ml non-Luer lock syringe was used to scrape the coral surface while simultaneously aspirating the resulting dislodged coral mucus and tissue. Colonies without lesions (control mucus+tissue slurry) were sampled along with colonies with lesions at the lesion boundary (diseased mucus+tissue slurry). Samples were frozen at −80 °C or in liquid nitrogen vapors until analysis.

### Preparation of nucleic acids

Tissue-only samples were obtained by decalcifying the skeleton, similar to previous studies [24, 41]. For this, frozen tissue was thawed, preserved in 4% paraformaldehyde, and placed in a 20% ethylenediaminetetraacetic acid (EDTA) solution at pH 7.8, changed daily, on a gentle rocker at 4 °C until skeleton was fully dissolved [24]. Due to the multiple rinsing, mucus is no longer observed by the end of this process. Following methods used by Apprill et al. [24], total DNA was extracted from swabs of mucus and decalcified coral tissue, with additional enzyme and high heat exposure. DNA was extracted from all sample types using the DNeasy PowerBiofilm DNA isolation kit ﻿(Qiagen, Valencia, CA, USA) following manufacturer instructions with some modifications (See Supplementary Methods). For the February 2020 field samples, DNA extraction, PCR, and sequencing methods are detailed in Becker et al. [22]. Nucleic acids from all sample types were eluted in 100 µl of buffer and stored at −20 °C.

### PCR and sequencing

Bacterial and archaeal small subunit ribosomal RNA genes were amplified from samples as well as 26 processing controls (consisting of unused swabs and empty bead beating tubes) using PCR, with reactions targeting the hypervariable IV region using primers modified for the marine environment [42, 43]. The 25-µl PCR mixtures were combined in an AirClean Systems 600 PCR Work Station (Creedmoor, NC, USA) and contained the following mixture: 14.75 µl DNA free water, 5 µl ﻿Colorless GoTaq Flexi buffer (Promega Corporation Madison, WI, USA), 2.5 µl MgCl_2_, 0.5 µl deoxynucleotide triphosphate (dNTP), 0.5 µl ﻿of each barcoded primer (515FY and 806RB) [42, 43], 0.25 µl goTaq Flexi DNA polymerase, and 1 µl of sample or control DNA. The mixtures were loaded into a 150 µl, 96 well plate (Eppendorf Corporation Hauppauge, NY, USA) and reactions conducted in a C1000 Touch Thermocycler (BioRad, Philadelphia, PA, USA). Initial PCR reaction conditions included: 95 °C heating step for 2 min, followed by 37–40 cycles (optimized for each sample) of 95 °C for 20 s, 55 °C for 15 s, 72 °C for 5 min, and then ending with a 72 °C step for 10 min. Gel electrophoresis was used to visualize the PCR product and for purification of the amplified products. Gels contained 1.5% agarose high melt/medium resolution buffer (High Desert Bioscience, Eligin, AZ, USA) combined with 1 µl of Sybr Safe DNA Gel Stain (Invitrogen, Carlsbad, CA, USA) per 10 ml of 1% Tris-Borate-EDTA solution. The 25 µl of PCR product was combined with 5 µl of 5x DNA Blue Loading Buffer (Bioline USA Inc., Taunton, MA, USA) and loaded into the gel alongside the 50-base pair HyperLadder. Gels were run at 110 V and 60 mAmps for approximately 120 min to allow for ample separation of bands. Gels were viewed on Ingenius 3 system (Syngene International Ltd., Bangalore, India) using program Ingenius 3 GeneSys and then excised with a sterile scalpel. Isolated gel bands were purified using MinElute Gel Extraction kit (Qiagen, Venlo, Netherlands) following manufacturer instructions.

Unique Nextera index primers (Illumina Corporation, San Diego, CA, USA) were attached to samples using a second PCR reaction. In a 96-well plate, 5 µl (200 nM) of two different barcoded primers were used to create a unique barcode combination in each well, and then combined with 18.5 µl of DNA free water, 10 µl GoTaq Flexi 5× Buffer, 5 µl MgCl_2_ (2.5 mM), 1 µl (200 µM) of dNTP, 0.5 µl of GoTaq DNA polymerase, and 5 µl of purified PCR product, for a total reaction volume of 50 µl per sample. Samples were subjected to a second round of PCR, with reaction conditions that included a 95 °C heating step for 3 min, followed by 8 cycles of 95 °C for 30 s, 55 °C for 30 s, and 72 °C for 30 s, and ending with a final 72 °C for 5 min. Products were then purified with a QIAvac 96 kit (Qiagen) following the manufacturer’s protocol. Samples were ﻿quantified using the Qubit 2.0 dsDNA HS fluorescence assay (Invitrogen) and diluted in Tris HCl to 5 nM assuming an amplicon length of 450 bp. Finally, samples were pooled to create a 5 nM library, and then the entire library was then diluted to 1 nM, and finally 90 pM. Libraries were sequenced on an iSeq 100 Sequencing System (Illumina Corporation, San Diego, CA) with a 5% spike in of 90 pM PhiX Control v3 to account for low base diversity.

### Sequence processing

Quality filtering, error estimation, dereplication, removal of chimeras, and selection of amplicon sequence variants (ASVs), or unique sequences, were performed with DADA2 v. 1.14.1 software [44], on forward reads only due to minimal overlap between the forward and reverse reads, using the filtering parameters: trimLeft = 19, truncLen = 150, maxN = 0, maxEE = 1, rm.phix = TRUE. The initial sequencing read length was 150 bp and after trimming the final length of the amplicons was 131 bp. Also in DADA2, taxonomy was assigned to ASVs using the assignTaxonomy function and the SILVA small subunit ribosomal RNA database v. 132 [45]. Confirmation or further resolution of the taxonomy of key ASVs was conducted by importing aligned sequences into the Coral Microbiome Database [46] using the program ARB [47]. Next the ASV table, taxonomy table, and metadata file were imported into Phyloseq v. 1.30.0 [48] for analysis. Only bacterial and archaeal sequences were examined in this analysis, and those identified as chloroplasts or mitochondria were removed. Samples with fewer than 5000 reads were removed from the dataset (Supplementary Table 1). Sequence data were deposited at NCBI SRA under accession PRJNA666222.

### Statistical analysis

A distance matrix for all samples was constructed on relative abundance data using the Bray–Curtis dissimilarity metric and a nonmetric multidimensional scaling ordination was used to visualize dissimilarity of microbial communities and to explore groupings of samples by species and health status. Permutational Analysis of Variance (PERMANOVA) conducted using the *adonis* function in *vegan* [49] with 999 permutations was used to test for significant differences between the microbial community of control, diseased, and exposed samples nested by species. Similarity percentage (SIMPER) analyses were conducted using PRIMER software (PRIMER-E Ltd, Plymouth, UK) [50] to test for similarity between experimental control and diseased microbial communities by coral species. To determine percent change, we took the overall similarity between controls and treatments by species and then calculated the relative change (*x*_2_ − *x*_1_)/*x*_1_ and then multiplying by 100 to convert to a percentage. Beta diversity dispersion was measured by calculating the distance to centroid separately for mucus and tissue samples grouped by health status of corals (control, disease exposed, diseased) with the *betadisper* function in *vegan* and significance was tested with a Wilcoxon rank sum test with Benjamin Hochberg correction for multiple testing.

To identify ASVs significantly enriched in diseased samples compared to control samples the differential test function of the Corncob v. 0.1.0 [51] R package was used. This applied a parametric wald test and multiple comparisons were accounted for with a Benjamini–Hochberg false discovery rate correction set to 0.05. ASVs that were enriched in two or more sample types were identified as potential disease indicator bacteria, and were searched using the NCBI tool Blastn [52] to identify complete sequence matches to samples from other studies.

## Results

Of the transmission experiment corals exposed to a diseased SCTLD colony, 100% of the *C. natans* and *O. annularis* colonies showed signs of SCTLD (i.e. had active lesions), whereas 87.5% of *Siderastrea siderea*, 75% of *P. strigosa*, 62.5% of *Porites astreoides*, and 37.5% of *M. cavernosa* showed signs of SCTLD (additional results available within Meiling et al. [39]). None of the control apparently healthy colonies developed lesions. Because disease status could not be confirmed for disease treatment samples that did not develop a lesion, they are categorized separately from diseased samples and referred to as “disease exposed (no lesion)”. There was an average of 70,453 reads (Supplementary Table 1) after quality filtering and trimming sequence reads per sample. Of these, there were 373 archaeal and 20,025 bacterial ASVs. Finally, controls had an average of 22,149 reads.

### Microbial community structure

**Fig. 2:**
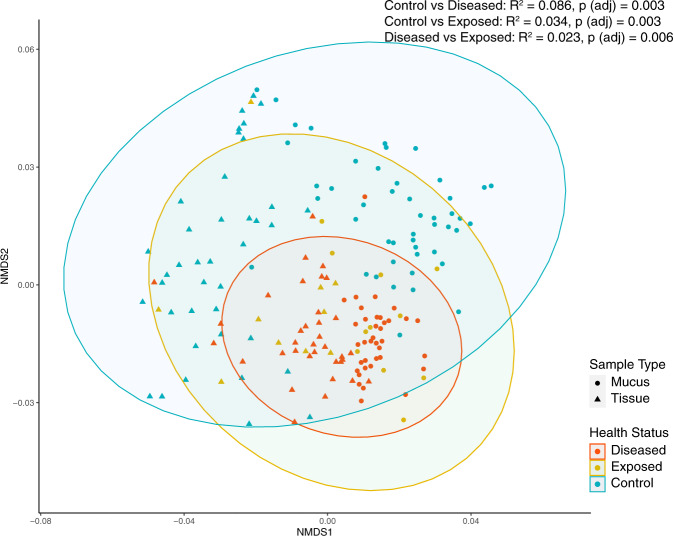
SCTLD lesioned coral microbiomes differ from disease exposed and apparently healthy (control) colonies. Nonmetric multidimensional scaling analysis of experimental mucus (circle) and experimental tissue (triangle) coral samples. Health status is differentiated by color (red = disease treatment, yellow = disease exposed (no lesions), and blue = control) and ellipses represent 95% confidence intervals. PERMANOVA results for significantly different microbial community composition by health status and *R*^2^ and adjusted p values are reported.

The multivariate analysis showed convergence of microbial communities based on disease status, regardless of coral microhabitat (mucus or tissue) and manipulation (experimental (Fig. 2)) or field (Supplementary Fig. 1). Additionally, there is high variability between samples, with clustering by sample type. A pairwise PERMANOVA test comparing the three health categories (control vs diseased, control vs disease exposed [no lesion], and disease exposed [no lesion] vs diseased) in the experimental colonies showed that health status had a significant effect on the coral-microbial community composition (Fig. 2, *R*^2^ = 0.0856, *p* = 0.003; *R*^2^ = 0.034, *p* = 0.003; *R*^2^ = 0.023, *p* = 0.006, respectively).

**Fig. 3:**
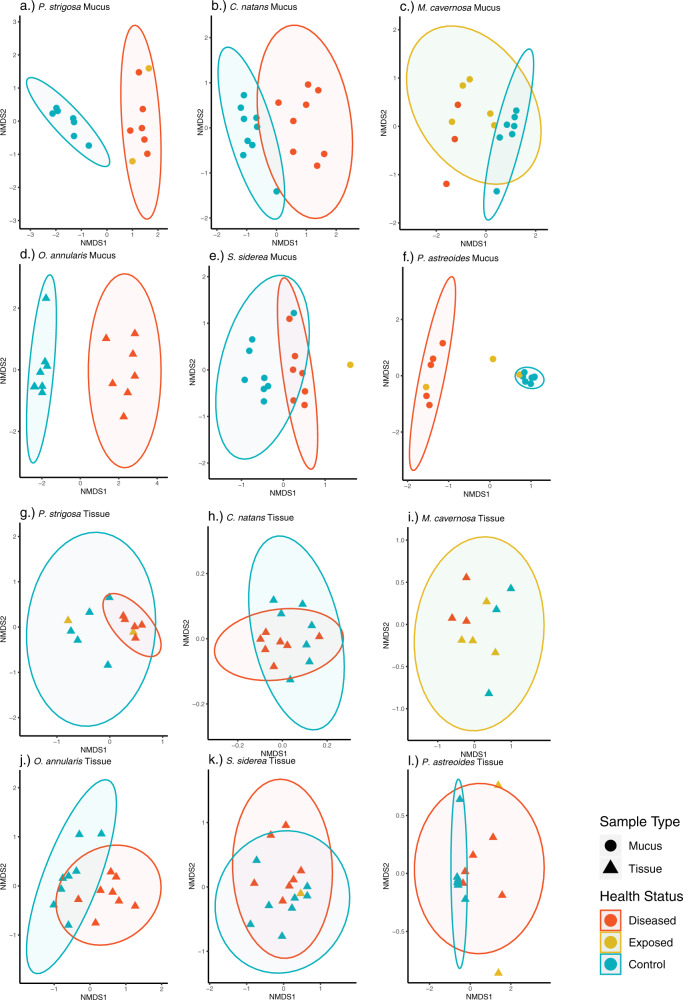
Species and microhabitat specific microbial response to Stony Coral Tissue Loss Disease. Mucus microbiome (**a**–**f**) exhibits shift in response to SCTLD, while tissue microbiome (**g**–**l**) remains more consistent. Nonmetric multidimensional scaling analysis of experimental mucus (circle) and tissue (triangle) samples. Health status is differentiated by color (red = disease treatment, yellow = disease exposed (no lesion), and blue = control) and ellipses represent 95% confidence intervals. Samples missing an ellipse had fewer samples than the four needed to calculate a confidence interval.

Examination of nMDS plots comparing Bray–Curtis dissimilarity microbiome data for each individual coral species by microhabitat highlighted the more pronounced difference in mucus microbial beta diversity with health status compared to that in tissue microbiomes, as well as variation in species-specific disease microbiome patterns (Fig. 3). For most species, both the mucus and tissue disease microbiomes were more variable compared to the “healthy” (experimental control and field-based) microbiomes. The PERMANOVA test demonstrated a difference in the microbial community of the coral mucus between the control and disease colonies within each coral species (Table 1, *R*^2^ = 0.15–0.57; *p* < 0.03). Additionally, mucus microbiomes of *P. strigosa*, *M. cavernosa* and *P. astreoides* differed between control and disease exposed (no lesion) colonies (*R*^2^ = 0.24–0.31, *p* < 0.4). In contrast, the control and disease (lesioned) tissue microbiomes of *P. strigosa*, *O. annularis* and *C. natans* significantly differed, *(R*^2^ = 0.16–0.23; *p* < 0.04), while those of *M. cavernosa, P. astreoides* and *S. siderea* did not (*R*^2^ = 0.08–0.33; *p* > 0.07). Dispersion of beta diversity was significantly different between control and diseased treatments in both mucus (Supplementary Fig. 2) and tissue (Supplementary Fig. 2) sample types, but not significantly different in control or disease compared to disease exposed (no lesion) treatments (Wilcoxon rank sum test *p* < 0.01). Distance to centroid was lower for disease treatments compared to controls, but overall, the values had greater variability between samples in disease treatment corals.

**Table 1:** Description of ADONIS statistical test examining the impact of lesion state on the mucus and tissue microbiomes of *P. strigosa, M. cavernosa, P. astreoides, S. siderea, O. annularis*, and *C. natans* (asterisk and bold indicated *p* < 0.05) for experimental mucus and experimental tissue from control vs diseased, control vs exposed (treatment corals that did not develop lesions), and exposed vs diseased corals.

	Control vs diseased		Control vs exposed		Exposed vs diseased	
Species	*R*^2^	*P* (adj)	*R*^2^	*P* (adj)	*R*^2^	*P* (adj)
Experimental mucus samples						
* P. strigosa*	0.320	0.009*	0.261	0.029*	0.125	1.000
* M. cavernosa*	0.330	0.03*	0.240	0.012*	0.200	0.123
* P. astreoides*	0.570	0.006*	0.310	0.036*	0.180	0.555
* S. siderea*	0.150	0.003*	0.180	0.360	0.190	0.414
* O. annularis*	0.380	0.002*				
* C. natans*	0.200	0.001*				
Experimental tissue samples						
* P. strigosa*	0.229	0.030*	0.162	1.000	0.220	0.423
* M. cavernosa*	0.330	0.300	0.220	0.435	0.210	0.140
* P. astreoides*	0.190	0.078	0.210	0.222	0.100	1.000
* S. siderea*	0.080	1.000	0.080	1.000	0.120	1.000
* O. annularis*	0.168	0.001*				
* C. natans*	0.160	0.011*				

**Fig. 4:**
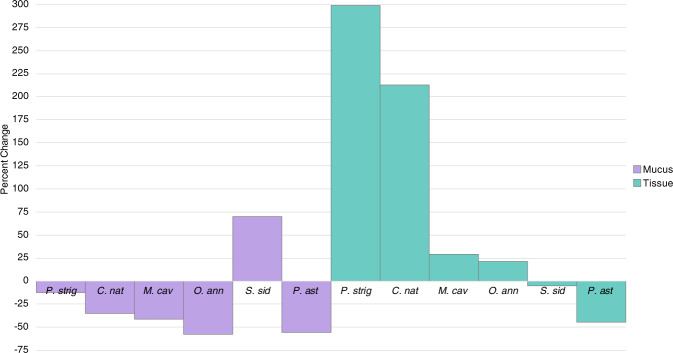
SIMPER results showing the percent changes in microbiome similarity patterns with SCTLD lesions. The percent change in the microbial community similarity between experimental control and treatment corals with active disease lesions in mucus (purple) and tissue (teal) compartments ordered from most to least susceptible species, calculated from SIMPER analysis. A positive value corresponds with an increase in similarity with disease state while a negative value corresponds with a microbial community that became less similar with disease acquisition. See Supplementary Fig.  3 for percent similarity within control and treatment groups.

To quantify the similarity in the microbial community composition among treatments, a SIMPER analysis was conducted on mucus and tissue microbiomes. The positive percent change between control and treatment colonies represents microbial community convergence and negative percentages represent divergence (Fig. 4). Generally, mucus microbiomes became less similar with disease, except for *S. siderea*. In contrast, the tissues generally converged (except for *P. astreoides)* upon disease acquisition. Interestingly, the most susceptible species, *P. strigosa* and *C. natans*, had the smallest divergence in the mucus microbiome (12% and 35% change, respectively) and the largest convergence in the tissue microbiome (percent changes of 300% and 212%, respectively).

### Disease bioindicator bacteria

To identify significantly differentially abundant ASVs in diseased compared to control corals, each experimental sample type (i.e., mucus and tissue) for each species was subset and analyzed using the corncob R package v. 0.1.0 [51] which applies a beta binomial regression model. Focusing on ASVs common between two or more species, the model identified a total of 43 ASVs enriched in disease samples (Table 2) and 26 enriched in healthy samples (Supplementary Table 2), some of which were enriched in multiple coral species. ASVs enriched in diseased samples were identified as part of the following Families: Arcobacteraceae, Cellvibrionaceae, Clostridiaceae, Colwelliaceae, Cryomorphaceae, Cuclobacteriaceae, Desulfovibrionaceae, Family XII, Flavobacteriaceae, Hyphomonadaceae, Marinifilaceae, Nitrincolaceae, Peptostreptococcaceae, Prolixibacteraceae, Puniceicoccaceae, Rhodobacteraceae, Rubritaleaceae, Saprospiraceae, and Vibrionaceae. Additionally, the relative abundance of these disease indicators was examined for field samples (Supplementary Figure 3), which showed five ASVs that were ubiquitously present across all diseased sample types and microhabitats (Rhodobacteraceae ASVs 12, 15, 59, 75, 84 and Vibrionaceae ASV 59).

**Table 2:**
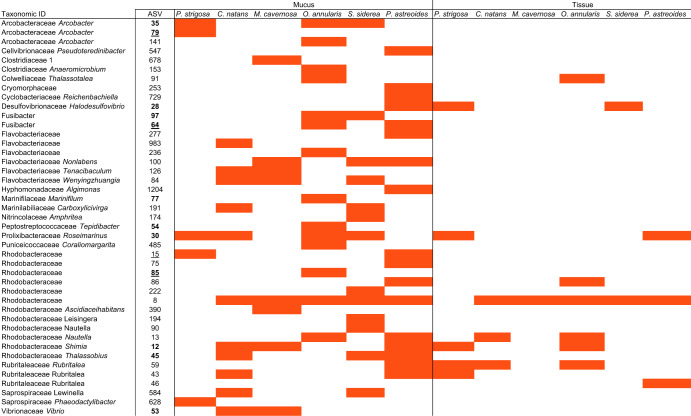
Summary of disease indicator bacteria, with ASVs in red significantly differentially enriched (*p* value < 0.05) in disease samples compared to apparently healthy controls for each species and microhabitat (i.e. mucus and tissue) based on corncob analysis.

Underlined ASVs indicate enrichment in *P. strigosa* and *O. annularis* mucus and tissue field samples collected in 2019 and bolded ASVs indicate enrichment in 2020 *M*. cavernosa and *C. natans* mucus + tissue slurry samples.

**Fig. 5:**
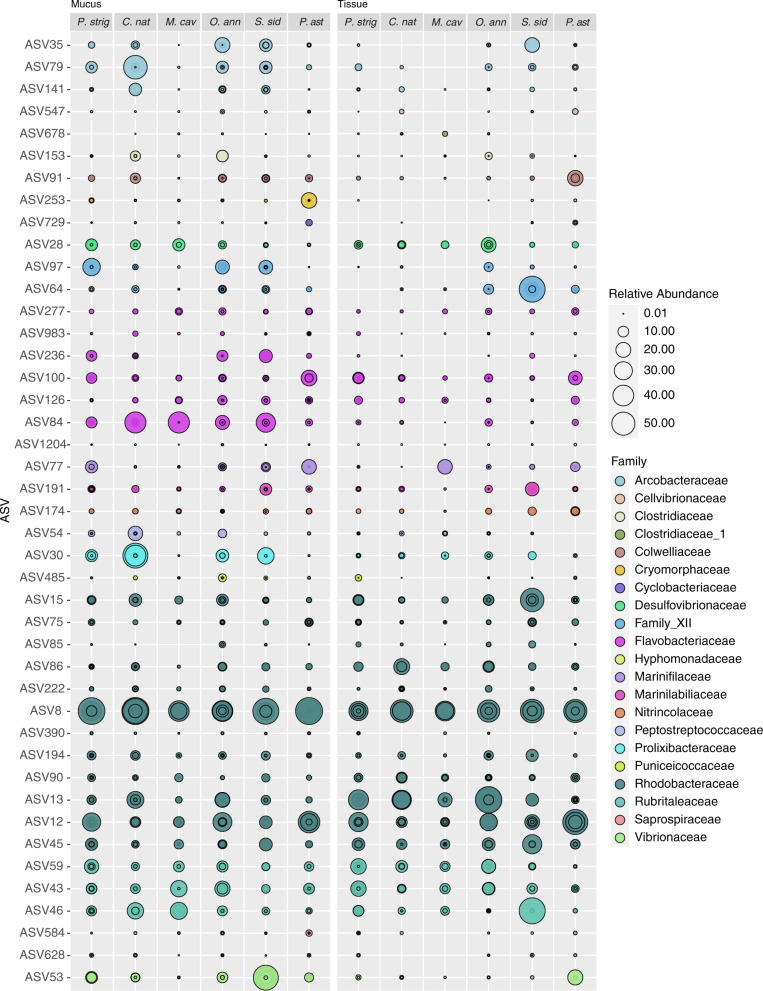
Relative abundance of amplicon sequence variants (ASVs) enriched in disease coral samples based on corncob analysis and colored by genus. Replicates for each sample type are overlain with each circle representing a replicate showing variability between samples.

There was no single ASV that was significantly enriched in disease samples across all coral species or microhabitats based on the corncob analysis, and thus no clear causative pathogen. However, from a presence/absence focus we identified many ASVs that although not identified as significantly enriched, were still present in the diseased samples (Fig. 5). For example, Rhodobacteraceae (ASV 8) was enriched within diseased corals in all species and microhabitats except for *P. strigosa*, yet had a relative abundance >40% in diseased *P. strigosa* mucus and tissue compartments. Notably, there were 28 ASVs that were present in the diseased microbiomes across all species and all sample types (experimental mucus and tissue), regardless of enrichment status.

Diseased tissue samples showed fewer enriched ASVs (10 total) compared to diseased mucus samples (42 total) (Table 2). Tissue samples were enriched in *Thalassotalea*, *Halodesulfovibrio*, *Roseimarinus*, two unidentified Rhodobacteraceae as well as *Nautella* and *Shimia*, and three *Rubritalea*. Mucus samples had these same taxonomic groups along with: *Arcobacter*, Cellvibrionaceae, Clostridiaceae, Cryomorphaceae, Cylobacteriaceae, Flavobacteriaceae, Hyphomonadaceae, Marinifilaceae, Nitrincolaceae, Peptostreptococcaceae, Puniceicoccaceae, Saprospiraceae, and Vibrionaceae.

### Sequence comparison

Blastn [52] was used to examine identical SSU rRNA gene sequences from public databases which matched to the 43 disease indicator bacteria sequences identified (Supplementary Table 3). Several ASVs were exact sequence matches to ASVs found in other coral disease studies associated with a diverse assemblage of coral species from various oceans, such as black band disease, white plague disease, white syndrome, white patch syndrome, heat-stressed coral, and white band disease.

BLASTn was also used to specifically compare these disease indicator bacteria to currently published SCTLD studies. These studies all used the mucus+tissue slurry collection method described here to sample corals in the field and sequenced the V4 regions of archaeal and bacterial SSU rRNA gene. Becker et al. [22] described the same St. Thomas, USVI mucus+tissue slurry samples used in this study, but with the addition of *Meandrina meandrites* and *Orbicella franksi*. The other two studies, Meyer et al. [29] and Rosales et al. [21], described microbiomes associated with diseased field samples in Florida. Sixteen ASVs were identified as exact sequence matches to the Becker et al. [22], Meyer et al. [23], and Rosales et al. [21] studies. Of the 16 ASVs identified as matches, 11 were found in the study by Becker et al. [22] [*Shimia* (ASV 12), Halodesulfovibrio (ASV 28), *Arcobater* (ASV 35 & 79), *Thalassobius* (ASV 45), *Tepidibacter* (ASV 54), Fusibacter (ASV 64 & 97), *Marinifilum* (ASV 77), Rhodobacteraceae (ASV 85), and *Vibrio* (ASV 53)]; one ASV identified as *Vibrio* (ASV 53) was also found in Meyer et al. [23], and three ASVs -- *Thalassobius* (ASV45) and two unidentified Rhodobacteraceae (ASV 75 & 390) were in common with the study by Rosales et al. [21] (Supplementary Table 3).

## Discussion

We applied a transmission experiment approach complimented with a subset of field samples, to examine responses of six species of coral to SCTLD. This is the first disease study to separate the mucus and tissue microbiomes of SCTLD infected corals. Mucus microbiome alterations were detected in both diseased (with lesion) and disease exposed (but without a visible lesion) colonies, suggestive of a mucus microbial response to SCTLD that occurs prior to visible lesions. In contrast, diseased tissue microbiomes showed differential species responses that followed species disease susceptibility. Microbiome similarity patterns among colonies also differed between the mucus and tissue compartments upon contracting the disease, which could relate to differences in the role of the mucus and tissue microorganisms in SCTLD. Lastly, we identified common disease-associated bacteria that may serve as indicators for SCTLD, 16 of which were identified in other SCTLD studies.

### SCTLD mucus microbiomes

Here we observed a significant shift in the diseased mucus microbial community of all species compared to apparently healthy controls, along with a community composition that changed to a less similar make up (except for *S. siderea*). No other study has examined mucus microbiomes separately from tissue in SCTLD affected corals and doing so allowed us to uncover opposing patterns in mucus and tissue microbiome similarity. The most susceptible coral species showed the smallest divergence in the mucus microbiome, which suggests that dysbiosis is occurring in this mucus layer and may be affecting the immune function provided by coral mucus, and that the most susceptible species are the most sensitive to loss of this protection offered by the mucus.

Our experiment showed novel trends in the mucus microbiomes of some SCTLD exposed (without lesion) colonies. Three species, *P. strigosa, M. cavernosa* and *P. astreoides*, showed significant differences in the mucus microbiomes of SCTLD exposed colonies, compared to controls, while *S. siderea* did not. While our observations bring up an interesting idea that the mucus microbiome could serve as an early diagnostic indicator of SCTLD exposure, more time-series type research is needed on susceptible colonies to conclude whether these genets were resistant to SCTLD, or in the early transitional period before lesion development. For future work, it would be informative to pair mucus sampling with histological investigations, to determine if the mucus microbiomes coincide with any of the observed histological changes in the coral tissue [39].

### SCTLD tissue microbiome alterations followed disease susceptibility patterns

In this study, the tissue microbiotas of the SCTLD lesioned colonies converged on a general disease signature (Fig. 2, NMDS). Half of the species, *P. strigosa, O. annularis* and *C. natans*, showed significant differences in their tissue microbiotas between lesioned and control colonies. These three species also showed increased similarity in the tissue microbiomes in the lesioned compared to control colonies. These results suggest that the associated microbes may be causative or highly reflective of the disease state in *P. strigosa, O. annularis* and *C. natans*. In contrast, *P. astreoides, S. siderea* and *M. cavernosa* lesioned tissue microbiomes were not significantly different than controls. These microbiome similarity patterns are reflective of SCTLD susceptibility patterns. Based on results from Meiling et al. [39] and in corroboration with the SCTLD case definition from the Florida Department of Environmental Protection [9, 53], the two most susceptible species in this study were *P. strigosa* and *C. natans*. Interestingly, these two species had the greatest percent change (300 and 212% respectively, Fig. 4) in the tissue microbial community with disease. The intermediately susceptible species included *M. cavernosa* (29%) and *O. annularis* (21%), which had a more moderate convergence in the tissue microbiomes compared to the two highly susceptible species. *S. siderea* is also an intermediately susceptible species, however, we found a slight divergence in the tissue microbiome. One caveat to consider is that there is uncertainty about whether or not *S. siderea* in fact contracts SCTLD based on differences in lesion morphology and progression [53]. However, based on the rapid lesion progression rates as well as no control colonies becoming diseased, we believe that *S. siderea* did in fact contract SCTLD in this study, and this species-specific microbial response may partly explain why the lesions are so different (Supplementary Fig. 4).

*P. astreoides* is considered a rarely susceptible species, but in this study, over half of the treatment colonies developed lesions by the end of the experiment. Notably the tissue microbial community behaved differently compared with the other species, diverging to a less similar community with disease. The high rates of infection in this study may be due to high pathogen loads within an enclosed system. Healthy *P. astreoides* tissue and mucus microbiomes in this study were highly similar (>65% among colonies, Supplementary Figure 5) and enriched with *Endozoicomonas* (Supplementary Table 2), which were lost upon lesion acquisition. Additional research is needed to understand why *P. astreoides* is more susceptible to SCTLD transmission in a lab setting, and if *Endozoicomonas*, prominent symbionts of *P. astreoides* [24, 54] provide protection against SCTLD in the field.

Conceptually, our results demonstrating microbiome change alongside coral health alterations align with the idea that the mucus microbiome serves as a primary immune response system for corals [26, 55]. Further, by showing microbiome divergence between colonies of each species (except *S. siderea*), our results suggest that there are likely several factors influencing SCTLD mucus microbiomes. For example, the alterations in the mucus microbiomes upon lesion development likely reflect primary and/or secondary infections, as well as potential reductions in beneficial microbes that contribute to host immune protection. The mucus and tissue microbial responses considered together suggest the microbiome responses among species are tied to species susceptibility, with the most susceptible species having the least dramatic divergence in the mucus microbiome and the most dramatic convergence to a very similar disease community, and the least susceptible species having a less similar tissue microbiome. This suggests that the shift to a dysbiotic community in the mucus microbiome results in a loss of the protective functions of the mucus and allows the tissue microbial community to become dominated by pathogens.

### SCTLD indicator bacteria

This laboratory-based experiment allowed us to sample the lesions early, likely reducing the number of secondary and saprophytic colonizers, and the observation of microbiome consistency among diseased colonies was also observed in the field-based samples, and thus is not likely an artifact of the laboratory setting (Supplementary Figs. 1 and 5). There were multiple bacteria that were exact sequence matches or similar (>97%) to those found in SCTLD field studies in the USVI and Florida, including multiple Rhodobacteraceae, Arcobacteraceae, Desulfovibrionaceae, Peptostreptococcaceae, Fusibacter, Marinifilaceae, and Vibrionaceae. Uncovering exact sequence matches across time and geographically disparate locations that were present, even if not identified as statistically more abundant, in all species and sample types suggests an important role that these ASVs are likely playing in this disease. However, it is unclear if these bacteria are causative agents, secondary pathogens, or associated with some part of the tissue breakdown process. Rhodobacteraceae were the most common Family of bacteria enriched in disease mucus and tissue samples (12 total, Table 2) and are common associates of both healthy and diseased corals. Some Rhodobacteraceae metabolize dimethylsulfoniopropionate (DMSP), and it is possible that they are attracted to this or other osmolytes released during the tissue sloughing. The genus *Nautella* includes *Nautella italica* R11, a pathogen that causes bleaching of red macroalgae, by secreting compounds that inhibit photosynthesis and that aid in algal cell wall penetration [56, 57]. Additionally, genomics studies have found that *Vibrio* have several virulence associated genes, allowing them to deploy many different tools to attack coral and their algal symbionts, such as toxins that cause photoinactivation in Symbiodiniaceae and tissue damage to the coral [58]. These bacteria warrant further investigation when considered alongside results from Landsberg et al. [31] which suggest that SCTLD first affects the coral’s algal endosymbiont, Symbiodiniaceae, and is a result of toxicosis. *Arcobacter* was only found to be enriched in disease mucus samples but not tissue. *Arcobacter* are found in various coral diseases globally which may point to its potential as an opportunistic bacterium (Supplementary Table 3). Desulfovibrionaceae thrive in anoxic, sulfide-rich environments and are a key secondary pathogen in the polymicrobial Black Band Disease, where its role as a sulfate-reducing bacteria results in sulfide production causing coral tissue death [59, 60]. Similarly, this bacterium may be acting as a secondary pathogen in SCTLD infected corals. Peptostreptococcaceae and *Fusibacter* are both anaerobic bacteria, and Marinifilaceae are facultatively anaerobic, further supporting that there is a reduction in the oxygen availability at the disease lesion. Histopathological and transmission electron microscopy has provided evidence that SCTLD is a result of toxicosis [31] or viral infection [19]. In the context of our microbial data, we are unable to determine if the bacteria identified are causing the disease or are opportunistic to the altered lesion conditions.

## Conclusions

The present study is the first to examine diseased coral and tissue microbiome samples separately, and the first disease transmission experiment conducted on stony coral tissue loss disease infected corals from the US Virgin Islands. This approach allowed us to control for variation in the microbial community in response to environmental changes, and also to make comparisons between the same coral individuals in a control and disease state for six coral species. A similar response to SCTLD infection– a shift to a more dissimilar microbial community – was seen in coral mucus when corals became infected suggesting that the disease is causing dysbiosis in the mucus layer, potentially impacting the immune function. In contrast, we found a species-specific response in the tissue community that is reflective of SCTLD susceptibility patterns, with the most susceptible corals having the greatest shift, converging to a highly similar microbial community within diseased coral tissue. A strength of the current study was the separate investigation of the mucus and tissue microhabitats, and we suggest future studies continue to focus on these communities separately to gain a clearer picture of the changes occurring in the coral microbiome.

## Supplementary information

Supplementary Methods

Supplementary Legends

Supplementary Figure 1

Supplementary Figure 2

Supplementary Figure 3

Supplementary Figure 4

Supplementary Figure 5

Supplementary Table 1

Supplementary Table 2

Supplementary Table 3
