# Supplementary material for: Experimental transmission of Stony Coral Tissue Loss Disease results in differential microbial responses within coral mucus and tissue
Source: ISME Commun. 2022 May 30;2:46. doi: 10.1038/s43705-022-00126-3 (PMC9723713; doi:10.1038/s43705-022-00126-3)
Supplement: Supplementary file 1 — Supplementary Methods [file 43705_2022_126_MOESM1_ESM.docx]

Supplementary

Methods

Preparation of Nucleic Acids:

For coral fragments, we obtained a tissue-only sample by decalcifying the skeleton, and this process also dissolves mucus. For this, frozen tissue was thawed, preserved in 4% paraformaldehyde placed in a 20% ethylenediaminetetraacetic acid (EDTA) solution on a gentle rocker at 4°C until skeleton was fully dissolved, with EDTA solution changed daily. Following methods used by Apprill et al. (2016), total DNA was extracted from a) swabs of mucus, b) decalcified tissue, and c) slurry (mucus, tissue, skeleton, and seawater) samples. DNA was extracted from samples using the DNeasy PowerBiofilm DNA isolation kit ﻿(Qiagen, Valencia, CA, USA) following manufacturer instructions with some modifications. Mucus: To remove as much mucus from the swabs as possible, before following the manufacturer’s protocol, heated MBL (55°C for 10 minutes) and FB solutions were added to the cryovials containing the sample swab, briefly vortexed, and centrifuged at 13 000 x g for 1 minute. Swabs were then handled with sterile forceps to turn the swab end up and centrifuged again at 13 000 x g for one minute. Swabs were then removed and discarded, while the solution containing MBL, FB, and mucus were transferred to the provided bead tubes and the protocol was followed starting at the 65°C 5-minute incubation. Tissue: Alterations to the DNEasy protocol for tissue samples included adding four additional UV-sterilized ceramic beads to the bead beating tubes to improve breakdown of the 0.05g of coral tissue, and an additional heating step at 90°C for one hour following the 5-minute incubation in the manufacturer’s protocols to depolymerize the paraformaldehyde. Both mucus and tissue samples had an extended (15-minute) bead beating step using a vortex mixer (Fisher Scientific, Stoughton, MA, USA) at full speed. Slurry (Mucus+Tissue): Samples were thoroughly vortexed for 10 seconds and 1.8ml of sample was transferred to a bead tube. Next samples were centrifuged at 12045 rcf for 10 minutes to concentrate the tissue and mucus and allow for the removal of the supernatant. Nucleic acids from all sample types were eluted in 100 µl of buffer and stored at -20°C.
