# Supplementary material for: Experimental transmission of Stony Coral Tissue Loss Disease results in differential microbial responses within coral mucus and tissue
Source: ISME Commun. 2022 May 30;2:46. doi: 10.1038/s43705-022-00126-3 (PMC9723713; doi:10.1038/s43705-022-00126-3)
Supplement: Supplementary file 2 — Supplementary Legends [file 43705_2022_126_MOESM2_ESM.docx]

Supplementary Figures

Supplementary Figure 1.

SCTLD lesioned coral microbiomes differ from disease exposed and apparently healthy (control) colonies. ﻿Nonmetric multidimensional scaling analysis of field mucus (circle), field tissue (triangle), experimental mucus (square), and experimental tissue (plus) samples. Health status is differentiated by color (red = disease treatment, yellow = disease exposed (no lesions), and blue = control) and ellipses represent 95% confidence intervals. Adonis tested for significantly different microbial community composition by health status, regardless of sample type.

Supplementary Figure 2.

Dispersion of beta diversity represented by box and whisker plots shows lower distance to centroid and greater variability in disease treatments (red) compared to control (blue) in both mucus (circle) and tissue (triangle) fractions. Disease exposed (no-lesion, yellow) show a lower distance to centroid in tissue but an intermediate distance in mucus samples.

Supplementary Figure 3. Relative abundance of amplicon sequence variants (ASVs) enriched in field disease samples (field mucus, field tissue, and mucus+tissue slurry) based on corncob analysis and colored by genus. Replicates for each sample type are overlain with each circle representing a replicate showing variability between samples.

Supplementary Figure 4. Photos of *Siderastrea siderea* with lesions after exposure to Stony Coral Tissue Loss Disease.

Supplementary Figure 5. Simper analysis comparing the percent similarity of the microbial community composition by species and by sample type (ie. mucus or tissue) for control and disease treatment experimental samples.

Supplementary Table 1. Percent of reads remaining after removing mitochondria and chloroplasts from samples. Samples in red indicate those with less than 5000 reads remaining and were not used in analysis.

Supplementary Table 2. ASVs enriched in control samples (in blue) for each species and sample type in the disease transmission experiment.

Supplementary Table 3. Blastn comparison for SSU rRNA gene sequence matches (>97%) to the ASVs enriched in disease samples to those identified in SCTLD field sampling in St. Thomas, USVI and Florida, as well as a general search for exact (100%) sequence matches to previous studies.
