# Supplementary figures and images for: Experimental transmission of Stony Coral Tissue Loss Disease results in differential microbial responses within coral mucus and tissue
Source: ISME Commun. 2022 May 30;2:46. doi: 10.1038/s43705-022-00126-3 (PMC9723713; doi:10.1038/s43705-022-00126-3)

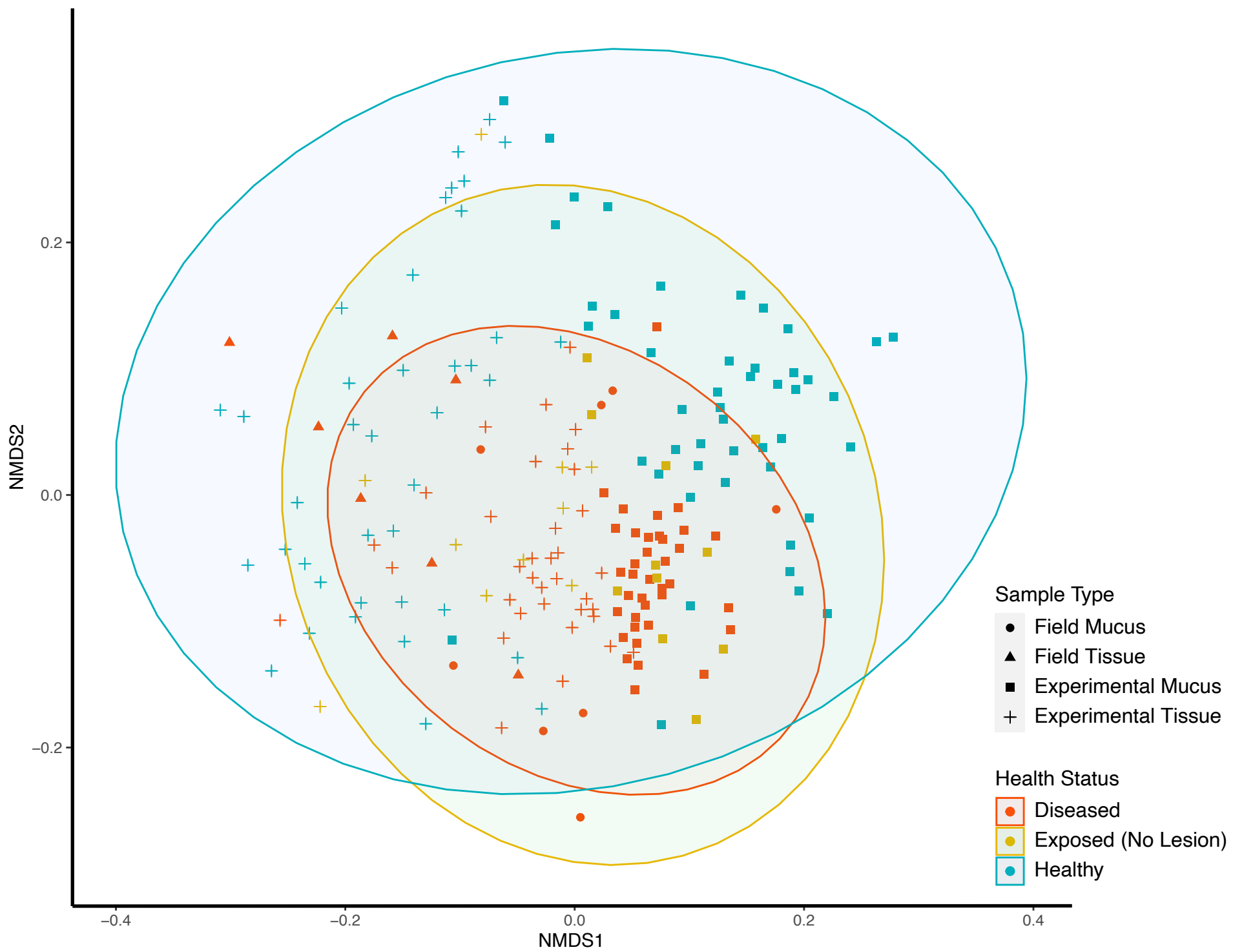

Supplement: Supplementary file 3 — Supplementary Figure 1 [file 43705_2022_126_MOESM3_ESM.pdf]

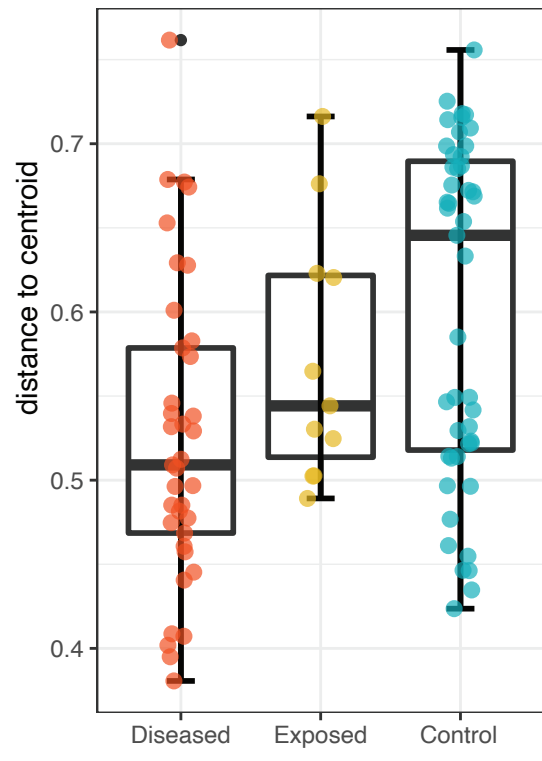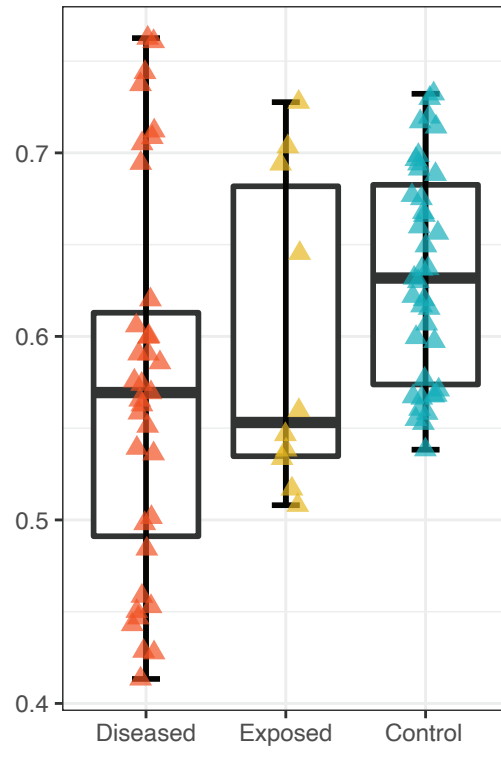

Health Status

- Diseased
- Exposed
- Control

SampleType

- Tissue
- Mucus

Supplement: Supplementary file 4 — Supplementary Figure 2 [file 43705_2022_126_MOESM4_ESM.pdf]

Relative Abundance of SCTLD lesion-associated ASVs across all corals

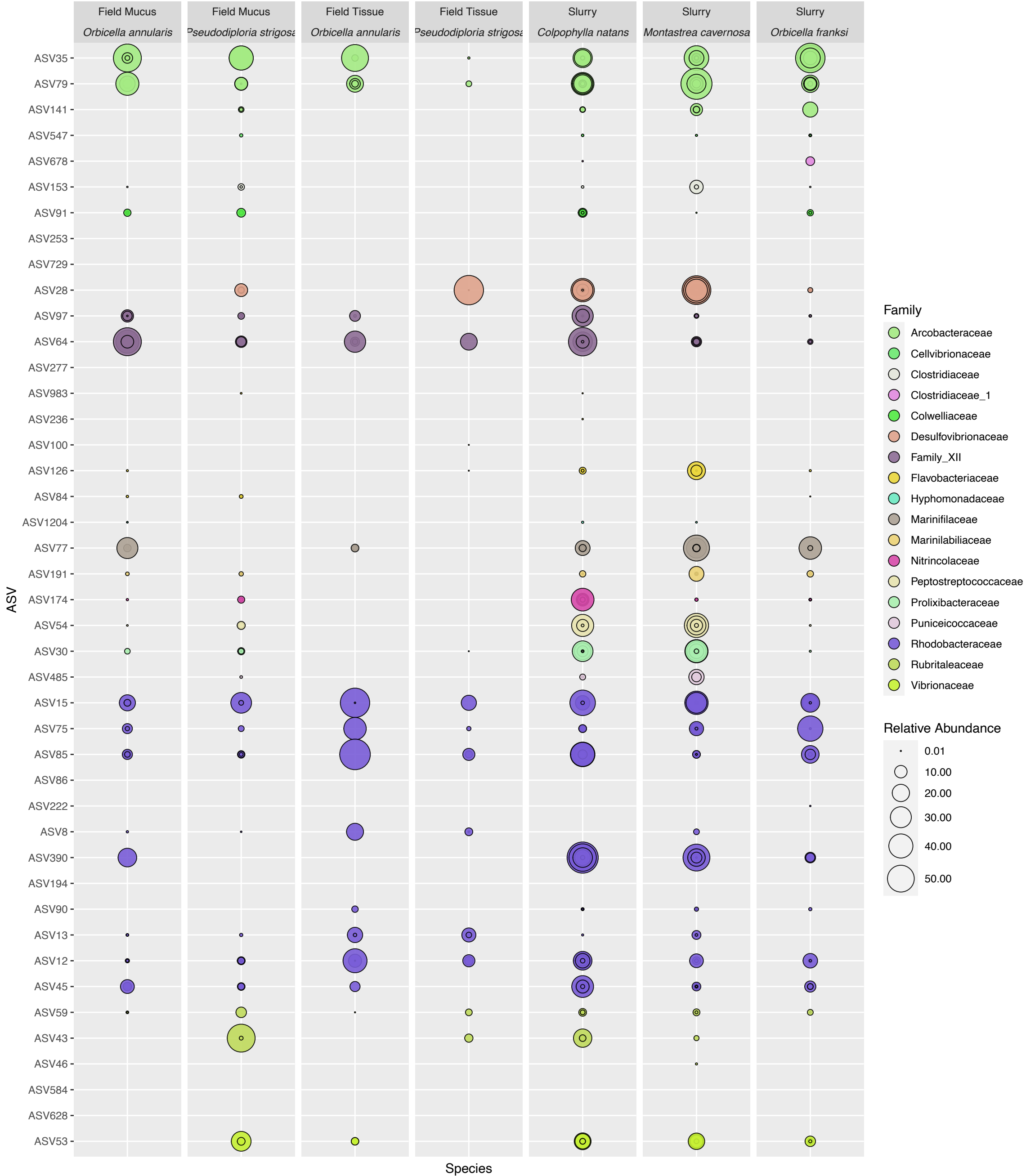

Supplement: Supplementary file 5 — Supplementary Figure 3 [file 43705_2022_126_MOESM5_ESM.pdf]

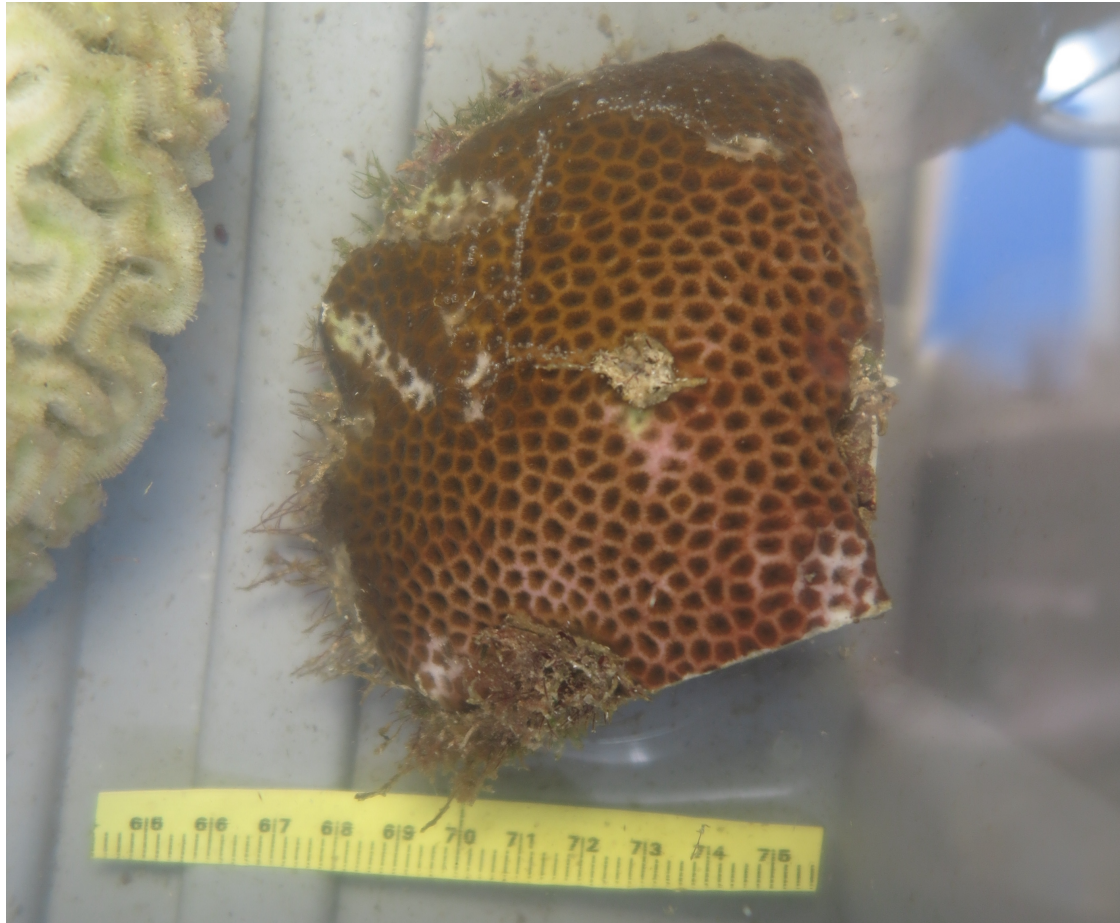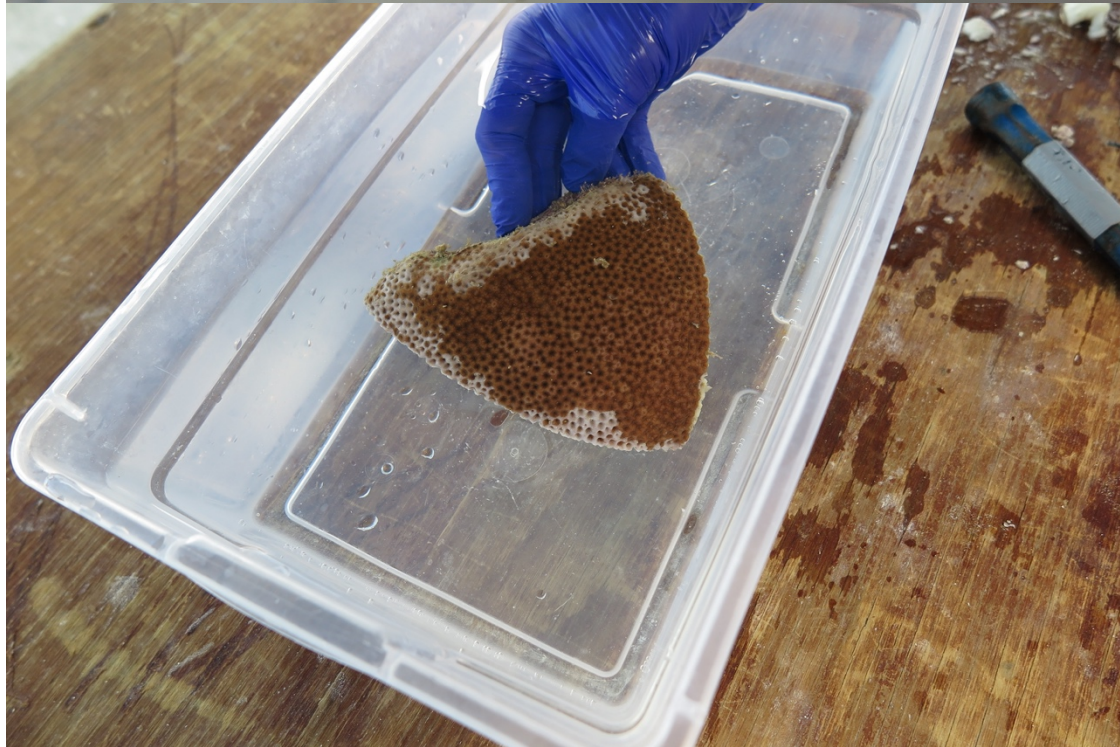

Supplement: Supplementary file 6 — Supplementary Figure 4 [file 43705_2022_126_MOESM6_ESM.pdf]

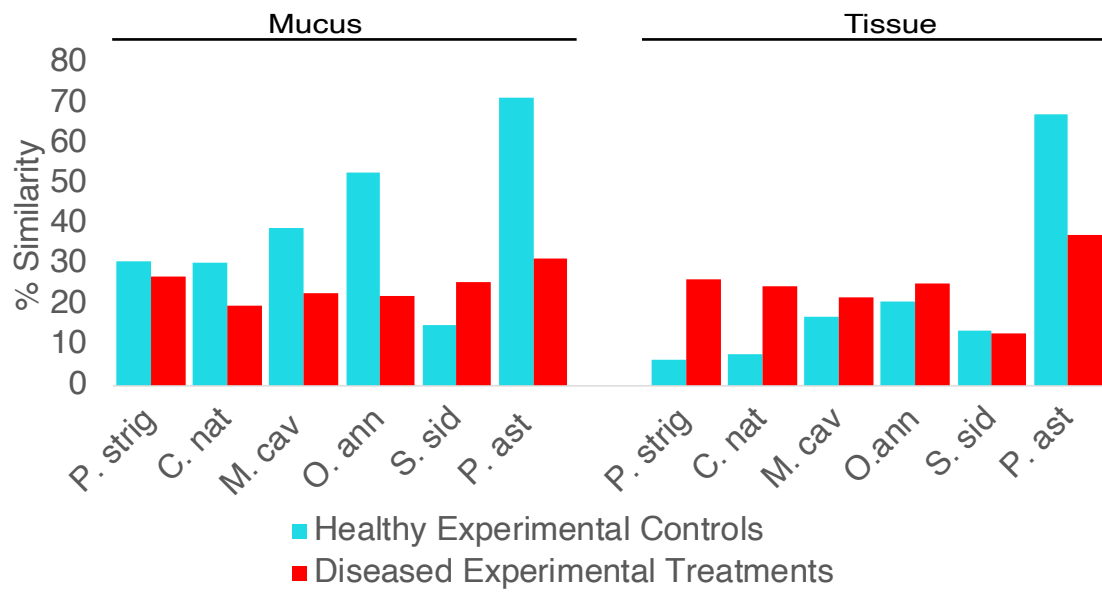

Supplement: Supplementary file 7 — Supplementary Figure 5 [file 43705_2022_126_MOESM7_ESM.pdf]
